# Pulmonary Hypertension in Aortic and Mitral Valve Disease

**DOI:** 10.3389/fcvm.2018.00040

**Published:** 2018-05-23

**Authors:** Micha T. Maeder, Lukas Weber, Marc Buser, Marc Gerhard, Philipp K. Haager, Francesco Maisano, Hans Rickli

**Affiliations:** ^1^Cardiology Division, Kantonsspital, St. Gallen, Switzerland; ^2^Department of Internal Medicine, Spital Rorschach, Rorschach, Switzerland; ^3^Department of Cardiovascular Surgery, University Hospital Zürich, Zürich, Switzerland

**Keywords:** pulmonary hypertension, post-capillary, pre-capillary, combined pre- and post-capillary, valve disease, aortic stenosis, mitral regurgitation

## Abstract

In patients with aortic and/or mitral valve disease the presence of pulmonary hypertension (PH) indicates a decompensated state of the disease with left ventricular and left atrial dysfunction and exhausted compensatory mechanism, i.e., a state of heart failure. Pulmonary hypertension in this context is the consequence of the backwards transmission of elevated left atrial pressure. In this form of PH, pulmonary vascular resistance is initially normal (isolated post-capillary PH). Depending on the extent and chronicity of left atrial pressure elevation additional pulmonary vascular remodeling may occur (combined pre- and post-capillary PH). Mechanical interventions for the correction of valve disease often but not always reduce pulmonary pressures. However, the reduction in pulmonary pressures is often modest, and persistent PH in these patients is common and a marker of poor prognosis. In the present review we discuss the pathophysiology and clinical impact of PH in patients with aortic and mitral valve disease, the comprehensive non-invasive and invasive diagnostic approach required to define treatment of PH, and recent insights from mechanistic studies, registries and randomized studies, and we provide an outlook regarding gaps in evidence, future clinical challenges, and research opportunities in this setting.

## Introduction

In patients with left heart disease, the presence of pulmonary hypertension (PH) is an important feature as it represents a marker of more advanced disease and poor prognosis (1). Pulmonary hypertension due to left heart disease (group 2 PH) is the by far most common type of PH, and valve disease is the leading cause (2). Independent of symptoms the presence of PH in patients with valve disease indicates a decompensated state of the disease with left ventricular (LV) and left atrial (LA) dysfunction and exhausted compensatory mechanism, i.e., a state of chronic heart failure (HF) with a propensity for acute exacerbations, e.g., in the context of arrhythmia or volume challenges. In the present article, we review the epidemiology and pathophysiology of PH in aortic and mitral valve disease and the diagnostic and therapeutic approach in these patients, and we provide considerations regarding future clinical challenges and research opportunities in this setting.

## Classification

Pulmonary hypertension in patients with left-sided valve disease is a consequence of valve disease and its impact on LV and LA function respectively and belongs to group 2 PH (3, 4). By definition this PH group is characterized by a mean pulmonary artery pressure (PAP, mPAP) ≥25 mmHg and a mean pulmonary capillary wedge pressure (mPAWP) >15 mmHg (4). In Table 1, a simplified version of the current PH classification (4) with the position of group 2 PH and in particular PH in valve disease is provided. Notably, group 2 PH is the only post-capillary form of PH. As discussed below the PH subgroups 2.1 to 2.3. cannot not be separated completely when discussing PH in the context of valve disease. As also discussed below there are patients who have two disease entities, i.e., valve disease and a form of PH not related to valve disease (i.e., non-group 2 PH).

**Table 1:** Classification of pulmonary hypertension (PH) [according to Galie et al. (3)].

**Group**	**Hemodynamic constellation**
**1 Pulmonary arterial hypertension**	**Pre-capillary** PH: mPAP ≥25 mmHg, mPAWP ≤15 mmHg
**2 PH due to left-sided heart disease2.1.** Left ventricular systolic dysfunction**2.2.** Left ventricular diastolic dysfunction**2.3.** Valvular disease**2.4.** Left heart inflow/outflow tract obstruction**2.5.** Pulmonary vein stenosis	**Post-capillary** PH: mPAP ≥25 mmHg, mPAWP >15 mmHg **Isolated post-capillary (IpcPH):** PVR ≤3 WU **Combined pre- and post-capillary (CpcPH):** PVR >3 WU
**3 PH due to lung disease and/or hypoxia**	**Pre-capillary** PH: mPAP ≥25 mmHg, mPAWP ≤15 mmHg
**4 Chronic thromboembolic PH and other pulmonary artery obstructions**	**Pre-capillary** PH: mPAP ≥25 mmHg, mPAWP ≤15 mmHg
**5 PH associated with unclear and/or multifactorial mechanisms**	**Pre-capillary and post-capillary** forms of PH

## Epidemiology

The prevalence of PH in patients with aortic and mitral valve disease depends on the type and severity of valve disease, the associated LV and LA dysfunction as well as other patient characteristics including age and cardiac rhythm and the method of PAP assessment. Direct measurement of mPAP by right heart catheterization (RHC), which is required for an exact diagnosis of PH, was performed in the minority of studies. The majority of data on the prevalence and prognostic impact of PH in valve disease are derived from echocardiographic studies where systolic PAP (sPAP) was estimated based on the peak tricuspid regurgitation velocity (TRV) using the Bernoulli equation. Many studies in the context of valve disease have defined significant PH as a sPAP ≥50 (TRV≈3.5 m/s) or ≥60 (TRV≈3.9 m/s) mmHg (Table 2). Independent of the presence or absence of additional echocardiographic signs of significant PH [such as right ventricular (RV) dilatation and/or dysfunction, D-shape of the left ventricle, shortened pulmonary acceleration time] these cut-offs are indicators of a high probability of PH and are associated with high specificity (4).

**Table 2:** Contemporary studies on the prevalence and prognostic impact of pulmonary hypertension (PH) in patients with aortic or mitral valve disease.

**Study**	**Age (years)**	**Severity of valve disease and setting**	**LVEF (%)**	**AF (%)**	**Pulmonary artery pressure**	**Main findings**
**Mitral stenosis (MS)**						
Fawzy et al. (5) (*n* = 531)	≈31	Severe MS: mean diastolic gradient ≈ 14 mmHgMVA≈0.8 cm^2^Balloon valvuloplasty in all patients	N.A.	≈13	Echo sPAP >60 mmHg: 15%	Worse 10 year event-free survival (redo valvuloplasty, mitral valve replacement) in patients with sPAP >60 mmHg than those with lower sPAP
Fawzy et al. (6) (*n* = 559)	≈31	Severe MS: mean diastolic gradient ≈ 15 mmHgMVA≈0.8 cm^2^Balloon valvuloplasty in all patients	N.A.	N.A.	RHC sPAP ≥50 mmHg: 38%	Normalization of sPAP (Echo) in most patients after a follow-up 4 years
Pourafkari et al. (7) (*n* = 558)	45 ± 13	Severe MS: Mean diastolic gradient 11 ± 6 mmHg, MVA 0.9 ± 0.1 cm^2^Balloon valvuloplasty in all patients	≈52	≈34	RHC or echo mPAP ≥25 mmHg: 81%	Very high prevalence of PH
Yang et al. (8) (*n* = 317)	61	MS: no further information, but all patients undergoing mitral valve surgery	N.A.	≈47	RHC or Echo sPAP 45–59 mmHg: 30% sPAP ≥60 mmHg: 40%	Worse long-term survival in patients with sPAP ≥45 mmHg than those with lower sPAP
**Mitral regurgitation (MR)**						
Ghoreishi et al. (9) (*n* = 873)	59 ± 14	Moderate (11%)Moderate-severe (24%) oder severe (65%) MRMitral valve surgery in all patints	52 ± 14	30	RHC (68%) or echo (32%) sPAP ≥50 mmHg: 32%	sPAP associated with operative and late mortality
Mentias et al. (10) (*n* = 1318)	62 ± 13	MR3+: ERO 0.56 ± 0.3 cm^2^86% of patients undergoing valve surgery	62 ± 2	18	Echo sPAP >50 mmHg: 15%	Prevalence of postoperative sPAP ≥35 mmHg: 19%Association between sPAP and mortality after follow-up of 7.1 years,
Barbieri et al. (11) (*n* = 437)	67 ± 11	MR (flail leaflet) grade 3–4: 95%Mitral valve surgery in 75% of patients	64 ± 10	24	Echo sPAP >50 mmHg: 23%	FU 4.8 yearsPH as long-term predictor of death and heart failure; mitral valve surgery beneficial but PH predictor of perioperative death
Le Tourneau et al. (12) (*n* = 256)	63 ± 12	MR grade 3 or 4 :ERO 51 ± 19 mm^2^Mitral valve repair or replacement in all patients	65 ± 10	29	Echo sPAP ≥50 mmHg : 32%	sPAP as independent predictor of mortality after a follow-up 4.1 years
**Aortic stenosis (AS)**						
Kusunose et al. (13) (*n* = 395)	70 ± 14	Moderate-severe or severe AS:MVG 38 ± 18 mmHgAVA 0.8 ± 0.2 cm^2^	59 ± 5	18	Echo sPAP 36 ± 11 mmHg	Follow-up 4.4 yearsNo association between sPAP and mortality
Lucon et al. (14) (*n* = 2435)	83 ± 7	Severe AS:MVG ≈48 mmHgAVA ≈0.7 cm^2^TAVR in 100% of patients	≈53	≈30	Echo sPAP ≥60 mmHg: 20%	Association between sPAP 40–59 and ≥60 mmHg and mortality after follow-up of one year
Urena et al. (15)(*n* = 3726)	81 ± 8	Severe AS:MVG ≈47 ± 17 mmHgTAVR in 100%	LVEF <40%:19%	30	sPAP >60 mmHg: 14%	Association between sPAP >60 mmHg and death due to heart failure after one year
Lindman et al. (16) (*n* = 542)	≈85	Severe AS:MVG ≈45 mmHg, indexed AVA ≈0.34 cm^2^/m^2^TAVR in 100%	≈52	≈37	Echo; sPAP ≈42 mmHgRHC: mPAP≈28 mmHg	Association between moderate and severe TR and RV/RA dilation and death but no significant association between sPAP and mortality
Bishu et al. (17) (*n* = 251)	81 ± 8	Severe AS:MVG 50 ± 13 mmHgAVA ≈0.8 cm^2^TAVR in 100% of patients	≈57	N.A.	Echo sPAP ≥49 mmHg: 33%	sPAP ≥49 mmHg associated with worse long-term mortality after median follow-up 328 days
O`Sullivan et al. (18) (*n* = 433)	≈83	Severe AS:MVG ≈42 mmHgAVA ≈0.6 cm^2^TAVR in 100% of patients	≈52	≈25	RHC mPAP ≥25 mmHg: 75%	Follow-up 1 yearAssociation between precapillary and combined pre- and postcapillary PH and mortality
Généreux et al. (19) (*n* = 1661)	≈83	Severe AS: MVG ≈44 mmHgAVA ≈0.7 cm^2^SAVR or TAVR in 100% patients	LVEF <50%:34%	40	Echo sPAP ≥60 mmHg; 27%	Association between sPAP ≥60 mmHg and/or moderate or severe TR and mortality after follow-up 1 year
Melby et al. (20) (*n* = 1080)	≈71	Significant AS (no further information)SAVR in 100% of patients	≈49	≈35	Echo or RHC sPAP ≥60 mmHg:9%	Association between sPAP ≥35 mmHg and higher mortality afterfollow-up 4 yearsBetter survival in those with pulmonary vascular resistance <3 WU than those with ≥3 WU
Nijenhuis et al. (21) (*n* = 591)	80 ± 8	Severe AS:MVG≈42 mmHg, AVA≈0.75 cm^2^TAVR in 100% of patients	≈55	39	Echo, probability of PH: low: 46%Intermediate: 22% high: 32%	High probability of PH as independent predictor of 30 days and 2 years mortality
Levy et al. (22) (*n* = 1019)	74 ± 11	Severe AS:MVG = 46 (35-58) mmHg,AVA = 0.76 (0.61–0.90) cm^2^75% with AVR (SAVR, TAVR)	63 (57–69)	31	EchoPeak TRV >3.4 (46 mmHg): 11%	Peak TRV >3.4 m/s as independent predictor of mortality after a median follow-up 31 months
Barbash et al. (23) (*n* = 415)	84 ± 8	Severe AS:MVG ≈48 mmHg,AVA ≈0.65 cm^2^TAVR in 100% of patients	53	≈42	Echo sPAP ≥50 mmHg: 59%	Higher 30 day and one year mortality in patients with sPAP ≥50 mmHg sPAP as independent predictor of one year mortality
Magne et al. (24) (*n* = 749)	74 ± 8	Severe AS:MVG: 48 ± 17 mmHg, AVA 0.69 ± 0.17 cm^2^SAVR in 91% of patient	72 ± 10	14	RHC mPAP >25 mmHg: 32% pre-capillary PH: 8%	PH as independent predictor of 30 day mortality and long-term mortality (mean follow-up 4.6 years,)
Lindman et al. (25) (*n* = 2180)	≈84	Severe AS:MVG ≈42 mmHg, indexed AVA≈ 0.35 cm^2^/m^2^TAVR in 100% of patients	≈55	NA	RHC mPAP ≥25 mmHg: 64%	Increased 1 year mortality in women with mPAP ≥35 mmHg, not in men
Franzone et al. (26) (*n* = 469)	82 ± 6	Severe AS:MVG: 44 ± 17 mmHg, AVA 0.7 ± 0.2 cm^2^TAVR in 100% of patients	54 ± 14	68	RHC mPAP = 48 ± 14 mmHg	mPAP as predictor of two year mortality in univariate but not multivariate analysis
Cam et al. (27) (*n* = 317)	≈73	Severe AS:AVA≈0.7 cm^2^SAVR in 47% of patients	≈50	≈30	RHC mPAP ≥25 mmHg: 47%	Lower 30 day and long-term mortality (mean follow-up 548 days) in patients with mPAP >35 mmHg undergoing SAVR versus those not undergoing surgerySimilar long-term mortality in patients with mPAP 25–35 mmHg and those with mPAP >35 mmHg when undergoing SAVR
Sinning et al. (28) (*n* = 353)	81 ± 7	Severe AS:MVG 42 ± 16 mmHg,AVA 0.7 ± 0.2 cm^2^TAVR in 100% of patients	48 ± 14	27	Echo: sPAP >60 mmHg: 26%	Higher 30 days and 2 year mortality in patients with sPAP 30–60 mmHg and sPAP >60 mmHgWorse prognosis in patients with persistent PH (sPAP >60 mmHg) after TAVR (Mean follow-up 517 days)
Ben-Dor et al (29) (*n* = 509)	≈82	Severe ASMVG ≈43 mmHg,AVA ≈0.7 cm^2^	≈50	N.A.	Echo≥60 mmHg: 34%	Association between higher sPAP and higher mortality after median follow-up 202 days
Masri et al. (30) (*n* = 407)	≈83	Significant AS: MVG ≈48 mmHg,AVA ≈0.6 cm^2^TAVR in 100% of patients	≈55	46	RHC mPAP ≥25 mmHg: 67%	Persistent at least moderate PH (Echo sPAP >45 mmHg) in 25%, which was an independent predictor of mortality
Testa et al. (31) (*n* = 990)	≈81	Severe AS:MVG ≈44 mmHgTAVR in 100% of patients	≈52	23	Echo sPAP >60 mmHg: 22%	Higher 1 year mortality in patients with baseline sPAP 40–60 mmHg and sPAP >60 mmHgPost-TAVR sPAP >60 mmHg at one month as independent predictor of 1 year mortality
D’Ascenzo et al. (32) (*n* = 674)	≈81	Severe AS:MVG ≈49 mmHg,AVA ≈0.6 cm^2^TAVR in 100% of patients	≈55	N.A.	Echo sPAP >40 mmHg: 47%	Higher 30 days and long-term (median follow-up 477 days) mortality in patients with sPAP >40 mmHG
Roselli et al. (33) (*n* = 2385)	74 ± 10	Severe AS:MVG = 48 ± 16 mmHg,AVA = 0.66 ± 0.14 cm^2^SVAR in 100% of patients	53 ± 13	12	Echo sPAP >50 mmHg: 24%	Higher in-hospital and long-term (mean follow-up 4.3 years) mortality in patients with higher sPAP
Schewel et al. (34) (*n* = 439)	80 ± 7	Severe AS (no further details)TAVR in 100% patients	53 ± 13	47	RHC mPAP ≥25 mmHg: 53%	Higher 30 days and one year-mortality in patents with mPAP ≥25 mmHg
**Aortic regurgitation (AR)**						
Khandhar et al. (35) (*n* = 506)	≈63	Severe AR	≈52	≈33	Echo sPAP ≥60 mmHg: 16%	32/83 patients with sPAP ≥60 mmHg undergoing surgery: better outcome than those not doing so

AF, atrial fibrillation, AVA: aortic valve area, AVR: aortic valve replacement, LVEF: left ventricular ejection fraction, mPAP: mean pulmonary artery pressure, MVA: mitral valve area, MVG: mean valvular gradient, NA: not available, RHC: right heart catheterization, sPAP: systolic pulmonary artery pressure, SVAR: surgical aortic valve replacement, TAVR: transcatheter aortic valve replacment, TRV: tricuspid regurgitant velocity.

In Table 2, important contemporary studies on the prevalence of PH in aortic and mitral valve are summarized (5–35). In patients with mitral stenosis (MS), 30–40% of patients undergoing valve replacement or valvuloplasty have substantial PH (systolic PAP > 50–60 mmHg by echocardiography or mean PAP > 40 mmHg by RHC). In series of patients with moderate or severe mitral regurgitation (MR) undergoing valve surgery early after the echo or later during follow-up in the majority of cases, a prevalence of PH defined as sPAP >50 mmHg was found in 15–32% of patients. For patients with aortic regurgitation (AR) there is very little data. One study found a prevalence of 16% of PH defined as sPAP 60 ≥ mmHg in a cohort of patients with severe AR (35). In patients with AS the prevalence of PH is high, particularly in the elderly. Up to 75% of patients with severe AS undergoing TAVR were found to have PH as assessed by RHC. In echo studies, the prevalence of sPAP >60 mmHg or ≥60 mmHg varied between 9 and 34%.

### Mechanisms of PH in Aortic and Mitral Valve Disease

With the exception of the relatively rare cases of patients with valve disease who have a second disease unrelated to valve disease and therefore do not have group 2 PH, the primary mechanism of elevated PAP in patients with left heart disease is the backward transmission of an elevated LA pressure (LAP) (3). The latter can be estimated by measurement of PAWP by RHC since direct access to the LA is not possible. It has to be realized however that there are some patients in whom PAWP does not reflect LAP: those with pulmonary veno-occlusive disease (high PAWP, normal LAP, obstruction of pulmonary venules) and those pulmonary vein stenosis (high PAWP, normal LAP). In MS and MR, valve disease has direct impact on the LA, whereas in aortic stenosis (AS) and AR the LA is affected indirectly by LV dysfunction. Many patients in whom criteria for severe AS, AR, or MR are clearly met, do not have an elevated PAWP however. The latter only occurs if the compensatory mechanism (LV and LA dilatation and function) are exhausted and/or if there is significant hypervolemia. Elevation of LAP is the result of LV systolic [reduced left ventricular ejection fraction (LVEF), reduced strain] and diastolic dysfunction (impaired relaxation and increased passive stiffness) and LA dysfunction (reduced compliance and booster function). Details of specific valve diseases are discussed below. A general principle however applies for the pathophysiology of PH in all types of left heart and left-sided valve diseases: initially PH is a purely passive phenomenon with a high LAP and PAWP but low transpulmonary gradient (difference between mPAP and mPAWP) and low pulmonary vascular resistance (quotient of transpulmonary gradient and cardiac output; isolated post-capillary PH, IpcPH). However, recurrent and chronic LAP elevation can cause alveolar stress failure and eventually pulmonary vascular remodeling with the development of a pulmonary vascular component of PH as reflected by an elevation of transpulmonary gradient and pulmonary vascular resistance respectively (combined pre-capillary and post-capillary PH, CpcPH) (1, 36, 37). Little is known about the exact mechanisms underlying this process; it is suspected that similar mechanisms and mediators play a role as in the pathobiology of pulmonary arterial hypertension (36, 37). Recent post-mortem data from well characterized patients with HF with reduced LVEF (HFrEF) and HF with preserved LVEF (HFpEF] without significant underlying or associated valve disease have revealed that pulmonary vascular remodeling is global but that the hemodynamic severity of PH is primarily related to remodeling of pulmonary venules (38). Similarly to other forms of PH valve-disease related PH can lead to RV dilatation and dysfunction and secondary tricuspid regurgitation but the clinical presentation can vary significantly (LV phenotype – the more common situation – versus RV phenotype) (36). In addition, we have to consider that the cardiac chambers and pulmonary circulation not only work in series (although we will in the following use such a schematic presentation) but that direct interactions on the ventricular and atrial level occur via septum. For instance, there is evidence from patients with MR that LV-RV interactions may play a role in the pathophysiology of RV dysfunction independently of PH (39).

## Diagnostic Principles

In this paragraph, the general principles of the diagnostic approach are discussed, whereas the specific aspects for each valvular lesion are discussed below in the respective sections. The two basic questions in this context are the following ones: First, is PH present? Second, is the valve disease and consecutive cardiac dysfunction sufficient to explain the presence and extent of PH?

### Non-Invasive Estimation of PAP

The estimation of PAP by echocardiography in patients with valve disease follows the usual principles as outlined in current guidelines (4): the probability of PH (i.e., mean PAP ≥ 25 mmHg) is low if peak TRV is ≤2.8 m/s and there are no indirect echocardiographic signs of PH (such as RV dilatation and/or dysfunction, D-shape of the left ventricle, shortened pulmonary acceleration time). The probability of PH is intermediate if peak TRV is ≤2.8 m/s but there are indirect signs of PH or if peak TRV is 2.9–3.4 m/s but without indirect signs of PH. The probability of PH is high if peak TRV is 2.9–3.4 m/s with indirect signs of PH or if peak TRV is ≥3.4 m/s regardless of indirect signs of PH. To be correct the central venous pressure as estimated from diameter and respiratory variability of the inferior vena cava has to be added to the transtricuspid pressure difference (as calculated from peak TRV) to calculate systolic PAP. However, this has not consistently been done in clinical studies, and even the current PH guidelines do not make absolutely clear how we should deal with this issue. An assessment of the RV and the tricuspid valve must be performed at the same time as RV function is an important prognostic predictor in various settings including left-sided valve disease (40).

### Differential Diagnosis of PH

If there is non-invasive evidence of PH, the underlying mechanism has to be assessed by multimodal imaging with echocardiography again being the key tool. The key question always is whether there is post-capillary or pre-capillary PH. The first step to address this is to assess the severity of the stenosis or regurgitation of the aortic and/or mitral valve. According to the current understanding of the grading of the severity of valve disease, only lesions fulfilling criteria for severe valve stenosis or regurgitation are hemodynamically significant in that they can cause LV dysfunction, LAP elevation and subsequently post-capillary PH. A description of the grading of AS/AR/MS/MR is beyond the scope of this review but can be found elsewhere(41). The next step is the assessment of LV systolic and diastolic function and semi-quantitative estimation of LVEDP and LAP (in relation to right atrial pressure) by echocardiography. If the severity of valve disease and LV dysfunction are likely to explain the presence and extent of PH, and the entire picture fits to a post-capillary form of PH, RHC can be deferred, treatment can be initiated, and a follow-up echocardiogram will help to understand whether the working hypothesis was appropriate (1, 36).

**Figure 1:**
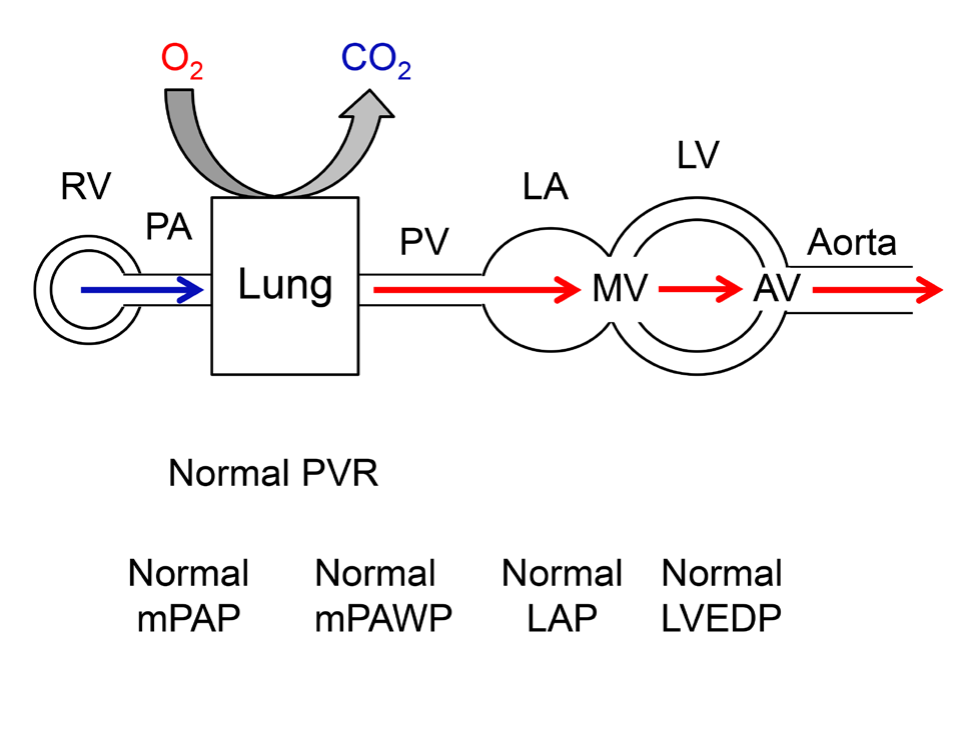
Normal hemodynamic situation of the circulation from the right heart across the lung and the left heart. AV: aortic valve, CO_2_: carbon dioxide, LA: left atrium, LAP: left atrial pressure, LV: left ventricle, LVEDP: left ventricular enddiastolic pressure, mPAP: mean pulmonary artery pressure, mPAWP: mean pulmonary artery wedge pressure, MV: mitral valve, PA: pulmonary artery, PV: pulmonary veins, PVR: pulmonary vascular resistance, O_2_: oxygen, RV: right ventricle.

**Figure 2:**
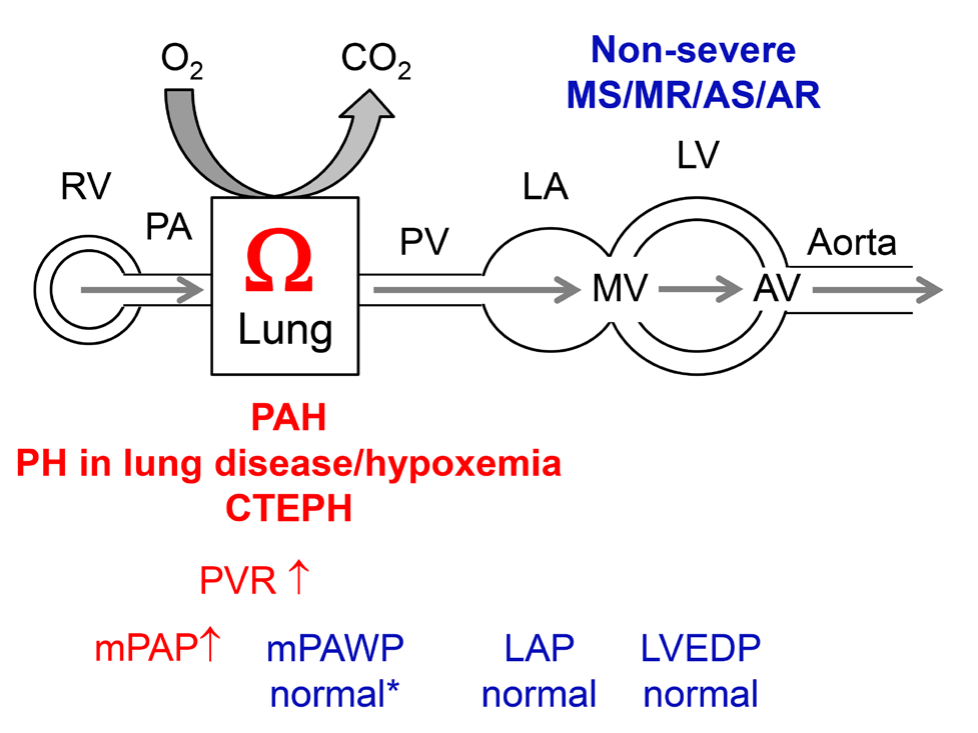
Hemodynamics of pulmonary hypertension (PH) other than group 2 PH in a patient with non-severe mitral stenosis (MS), mitral regurgitation (MR), aortic stenosis (AS), or aortic regurgitation (AR). PAH: pulmonary arterial hypertension, CTEPH: chronic thromboembolic PH. * typically relatively low. Other abbreviations as in Figure 1.

**Figure 3:**
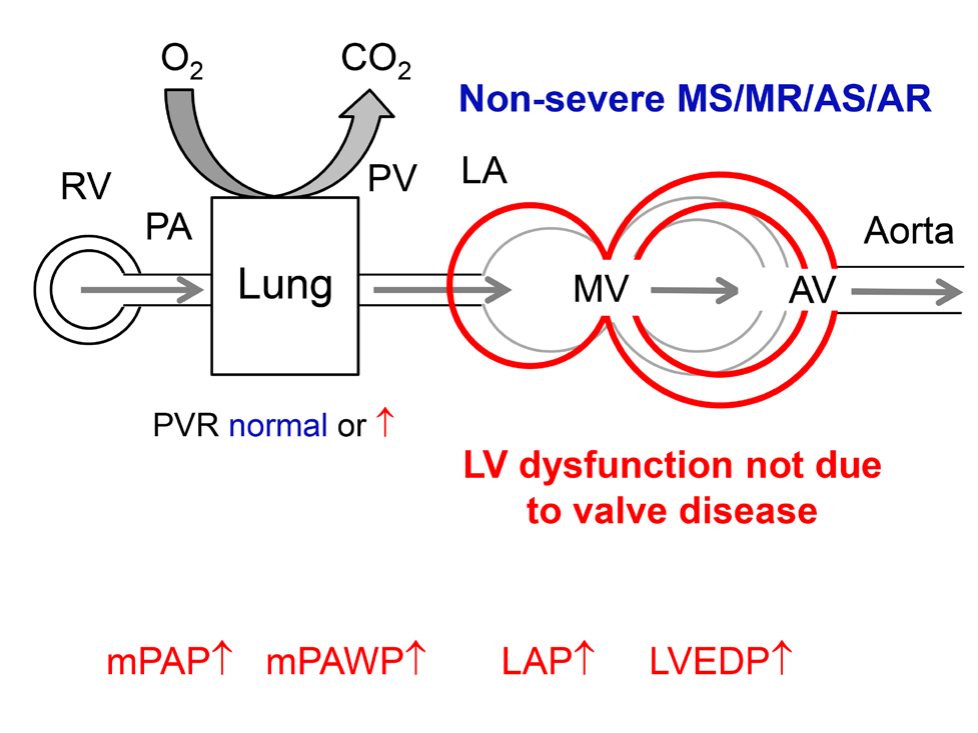
Hemodynamics of group 2 pulmonary hypertension due to non-valve disease related left ventricular (LV) dysfunction in a patient with non-severe mitral stenosis (MS), mitral regurgitation (MR), aortic stenosis (AS), or aortic regurgitation (AR). Other abbreviations as in Figure 1.

There will be situations however, where it remains unclear whether the presence and extent of PH is explained by the severity of valve disease. We will use in the following a diagram to present the various hemodynamic patterns in a simplified manner (Figure 1). The following scenarios have to be considered: a patient may have two diseases, i.e., either valve disease and non-group 2 PH (Figure 2) or valve disease and PH in the context of LV dysfunction which however is not primarily the result of valve disease (Figure 3) although valve disease can contribute to further progression of LV dysfunction. Examples for the latter include severely reduced LVEF due to non-ischemic cardiomyopathy and moderate secondary MR or moderately reduced LVEF after myocardial infarction and moderate AS. Echocardiography can help to distinguish between post-capillary (group 2 PH) and pre-capillary (non-group 2) forms of PH although this is not always reliable. A small or normal-sized left ventricle without hypertrophy, a high LV eccentricity index (i.e., a D-shaped LV), a larger right atrium than LA, and enlarged and apex-forming RV, a low peak early transmitral velocity to peak early mitral annular velocity (mitral E/e’), and a short pulmonary acceleration time are markers of pre-capillary rather than post-capillary PH (1).

**Figure 4:**
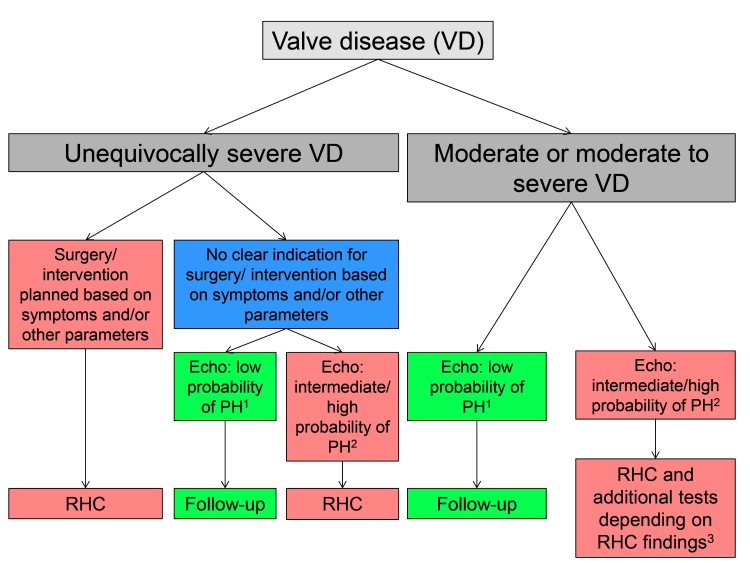
Suggested algorithm to detect pulmonary hypertension (PH) using echocardiography (echo) and right heart catheterization (RHC) in patients with left-sided valve disease (VD, i.e., mitral stenosis and/or regurgitation, aortic stenosis and/or regurgitation). ^1^low probability of PH: peak TRV ≤2.8 m/s and no indirect echocardiographic signs of PH ^2^intermediate probability of PH: peak TRV ≤2.8 m/s but indirect signs of PH or if peak TRV but without indirect signs of PH, high probability of PH: peak TRV 2.9–3.4 m/s with indirect signs of PH or peak TRV ≥3.4 m/s regardless of indirect signs of PH ^3^please see Figure 3.

In Table 3, there is a summary of clinical features which should raise the suspicion of the presence of non-group 2 PH or group 2 PH which is not the result of valve disease, which should trigger a more extensive work-up.. In these situations it will often be important to perform RHC to clarify the situation. Left heart catheterization with measurement of LVEDP will sometimes be helpful or even compulsory to unequivocally define the hemodynamic pattern and to exclude rare pathologies such as veno-occlusive disease. By definition (4) if there is PH (mPAP ≥25 mmHg), mPAWP must be ≥15 mmHg, and LVEDP must be similar, if PH is the result of MR, AR or AS. If PH is the consequence significant MS, mPAWP ≥15 mmHg and a significant diastolic PAWP-LV pressure gradient must be present. If mPAP is ≥25 mmHg but mPAWP is <15 mmHg, there is a non-group 2 PH. The presence of non-group 2 PH is relatively rare in patients with severe valve disease but its recognition is of paramount importance since in such a patient a mitral or aortic valve intervention will not lower pulmonary pressures. Having said that it is also well known that in some patients with longstanding CpcPH RHC may reveal pure pre-capillary PH after aggressive reduction of filling pressures by diuretics or and/or after valve replacement, and that only a volume challenge may reveal the true hemodynamic picture (42). In patients with severe valve disease and LV dysfunction likely to explain the presence of PH but non-invasive evidence of disproportionally high PAP, RHC will be important to assess whether there is IpcPH or CpcPH as this has prognostic impact (18). The current valve disease guidelines are relatively reluctant with regards to RHC during the work-up of a patient with valve disease potentially undergoing valve surgery/valve intervention (41). However, given the previous considerations we think that in patients evaluated/planned for valve surgery/intervention RHC should be performed at the time of the coronary angiogram. In Figure 4, an algorithm for the use of RHC in the assessment of a patient with valve disease and possible PH is shown.

**Table 3:** Clinical features in patients with valve disease and pulmonary hypertension (PH) suggesting the possibility of the presence of PH with a mechanism unrelated to valve disease; these considerations are particularly relevant if valve disease does not fulfil criteria for severe stenosis/regurgitation.

**Clinical context**	**Possible mechanism of PH**	**Diagnostic evaluation**
PH and significant hypoxemia (in absence of frank pulmonary edema)	PH due to lung disease and/or hypoxemia (group 3 PH)	Lung function, blood gas analysis, sleep studies, CT, right heart catheterization
PH and rheumatic disease (e.g., lupus erythematodes)	Group 1 PH	Rheumatology work-up, diffusion capacity, right heart catheterization
PH and non-severe valve disease in combination with normal LV size and function	Any non-group 2 PH	Right and left heart catheterization, search for other forms of PH (lung function, sleep studies, V/Q scan, rheumatology work-up)
PH and non-severe valve disease in combination with significant LV dysfunction	Any group 2 PH which is not (only) a consequence of valve disease (e.g., ischemic cardiomyopathy with moderate secondary MR)	Detailed echocardiography, cardiac MRI, Holter monitoring, left and right heart catheterization
PH and non-severe valve disease in combination with preserved LVEF and history of thoracic radiation	Group 2 PH in the context of HFpEF following radiation (significant coronary artery disease may also be present)	Detailed echocardiography, left and right heart catheterization
PH and non-severe valve disease, history of catheter ablation for atrial fibrillation years ago	Pulmonary vein stenosis	Left and right heart catheterization including measurement of LVEDP, CT
PH and non-severe valve disease, history of catheter ablation for atrial fibrillation years ago	Stiff left atrial syndrome	Left and right heart catheterization including measurement of LVEDP, cardiac MRI
PH and previous thrombosis/pulmonary embolism, immobilization, cancer, coagulation disorder	Chronic thromboembolic pulmonary hypertension (Group 4 PH)	V/Q scan, right heart catheterization

### Role of Exercise Hemodynamics

Although it is appealing to study PAP on exercise to discover the clinical relevance of valve disease in ambiguous situations (3, 43), the role of exercise PH is not well defined at the moment. Stress echocardiography studies have shown that exercise PH (typically defined as systolic PAP > 60 mmHg) is associated with the future occurrence of symptoms in asymptomatic patients with at least moderate degenerative MR (44), cardiac events in patients with secondary MR (45), and cardiac events in asymptomatic patients with severe AS (46). However, exercise PAP depends on pulmonary blood flow, and an absolute exercise s PAP value has to be interpreted in the context of exercise capacity and pulmonary blood flow respectively (3). In addition, exercise sPAP has been shown to be strongly related to resting sPAP (44–46), and therefore the additional information of exercise PH is somewhat limited. Furthermore, stress echocardiography cannot reveal the exact mechanism underlying the rise in sPAP during exercise (rise in LAP versus rise in pulmonary vascular resistance). Recently, it has become clear that PH is common also in HFpEF in absence of significant valve disease (37, 47) and there may be a spectrum of hemodynamic rest/exercise profiles ranging from an isolated rise in PAWP during exercise to a CpcPH pattern with a rise in both PAWP and PVR during exercise (48). A similar diversity is likely present in patients with valve disease but data on invasive exercise hemodynamics in patients with valve disease are lacking. Thus, guidelines (41) currently do not provide recommendations for interventions based on exercise PH.

### PH in Specific Valve Diseases

#### Mitral Stenosis

**Figure 5:**
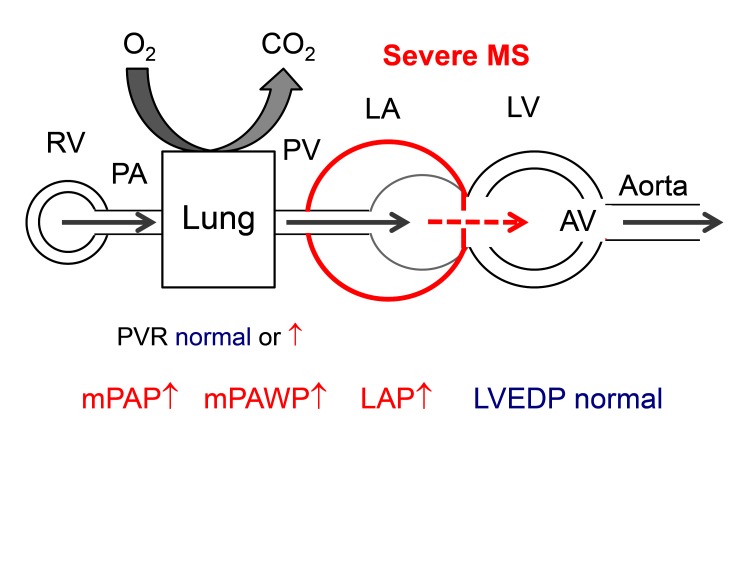
Hemodynamics of severe mitral stenosis (MS). It should be noted that the LV is not affected by the valvular problem, and LVEDP is normal. A significant diastolic gradient between LVEDP and PAWP is characteristic. Abbreviations as in Figure 1.

In patients with MS, the LV is not affected by the valvular problem although in rheumatic heart disease LV dysfunction can occur independently of valve disease. The obstruction at the level of the mitral valve leads to increased LAP which is directly backwards transmitted to the pulmonary veins (Figure 5) (3). The transmitral gradient is a strong but not the only determinant of PAP (49). Lower net atrioventricular compliance, a composite measure of LA and LV compliance, has been shown to be a predictor of sPAP independent of mitral valve area and mean diastolic pressure gradient (49). Notably, net atrioventricular compliance and mitral valve area have also been identified as independent predictors of valve intervention or death in patients with MS (49). The LA in severe MS is often significantly enlarged, and LA function is impaired due to fibrosis. This in turn can lead to atrial fibrillation which can further aggravate LA dilation and dysfunction.

Chronic LAP elevation can lead to pulmonary vascular remodeling and additional pre-capillary PH, and patients with long-standing severe MS often have substantial CpcPH. In a large study using RHC (*n* = 599), 38% of patients with severe MS undergoing percutaneous mitral commissurotomy had sPAP ≥50 mmHg. Many of these patients had CpcPH [mean PVR 329 dyn*sec*cm^−5^ (4.1 WU) and 857 dyn*sec*cm^−5^ (10.7 WU) in patients with sPAP 50–79 and ≥80 mmHg respectively] (6). This increase in RV afterload can lead to RV dilatation and dysfunction with secondary tricuspid regurgitation which can be further aggravated by structural alterations of the tricuspid valve in rheumatic heart disease. Studies have shown that significant pre-intervention PH is a predictor of poor long-term prognosis in patients with severe MS undergoing valve replacement or percutaneous mitral commissurotomy (5, 8). Isolated post-capillary PH usually resolves quickly after successful mitral valve intervention, whereas in patients with CpcPH, PH can persist despite normalization of LAP and PAWP, and paradoxically hemodynamics in such patients may reveal pure pre-capillary PH years after mitral valve intervention. Thus, incomplete resolution of PH with persistence of pre-capillary PH and RV dysfunction in patients with long-standing PH are the most likely mechanism underlying the prognostic value of PH in MS although one study has shown normalization of PH in all patients with PH even in those with baseline systolic PAP > 80 mmHg (6). The current ESC guidelines acknowledge the role of PH as a marker of more advanced disease and give a IIa recommendation for percutaneous mitral commissurotomy in patients with significant MS (valve area <1.5 cm^2^), no/little symptoms but a systolic PAP > 50 mmHg (41).

### Mitral Regurgitation

#### Primary Mitral Regurgitation

**Figure 6:**
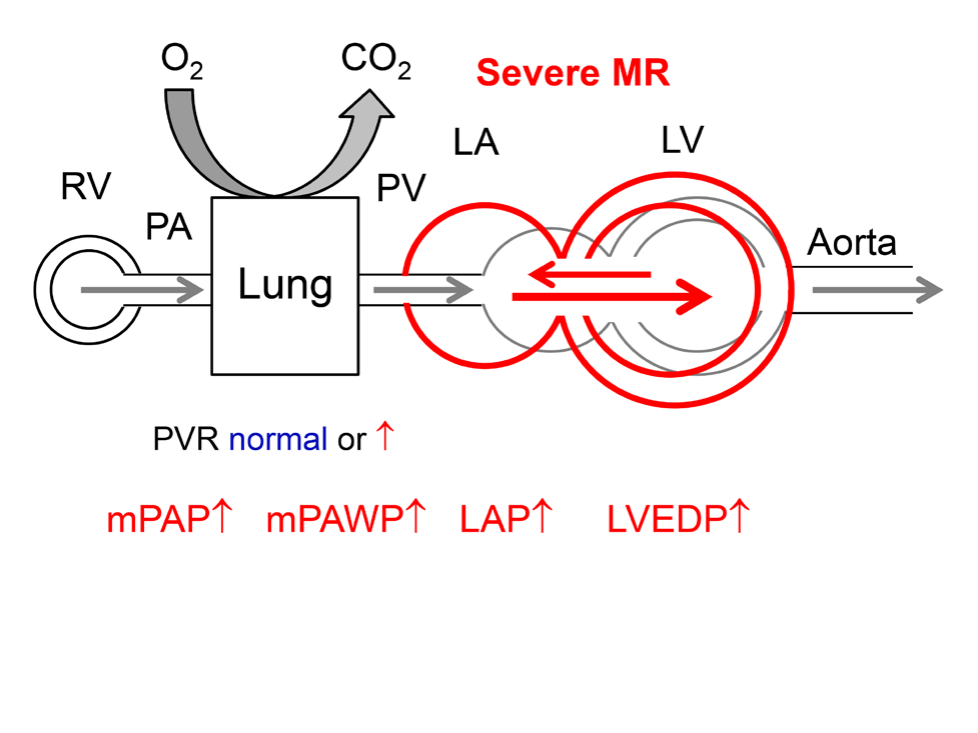
Hemodynamics of severe primary mitral regurgitation (MR). There is volume overload and dilatation of the both LV and LA. Abbreviations as in Figure 1.

The LV volume overload in chronic severe degenerative MR leads to progressive LV dilatation and eccentric hypertrophy and eventually systolic and diastolic dysfunction. This together with the direct effect of the systolic backflow into the LA under LV pressure can lead to LAP elevation with large V waves and PAWP elevation (Figure 6). The LV in severe primary MS is that of a special form of heart failure with preserved, midrange, or reduced LVEF. The ventricle is dilated, and as soon as LVEF is <60% there is systolic dysfunction which however is masked by the high preload (50). Any LVEF less than 60% in patients with degenerative MR is associated with reduced survival under medical therapy (51). Systolic LV dysfunction in MR is accompanied by increased LV stiffness, i.e., diastolic dysfunction. Left atrial enlargement is the result of LV dysfunction and volume overload and favors the occurrence of atrial fibrillation (52). Larger LA size in severe MR is associated with higher PAP and worse outcome (53). Independent predictors of higher sPAP in three studies included higher age (11), female sex (10), larger LA size (10–12), larger LV end-systolic dimensions (10), higher medial E/e’ (12), shorter mitral deceleration time (12) chronic lung disease (10), dialysis (10), previous myocardial infarction (10), and higher body mass index (10). In all large contemporary series of patients with MR with the majority undergoing surgery, baseline sPAP as assessed by echocardiography (3 studies) or RHC or echocardiography (one study) was a strong predictor of mortality (9–12).

Notably, despite successful mitral valve surgery, PH may persist. In a series of 1318 patients with degenerative MR with 86% undergoing mitral valve surgery, postoperative sPAP >35 mmHg was observed in 19% of patients and was predicted by older age and higher preoperative sPAP (10). In another large series (*n* = 873) of patients undergoing surgery for MR the sPAP in the entire population decreased from 43 mmHg before surgery to 39 mmHg early after surgery and remained unchanged after two years (9).

Both the 2017 ESC guidelines (41) and the 2017 update of the US guidelines (54) give a class IIa indication for surgery for asymptomatic patients with LVEF >60%, and LVESD <45 mm (i.e., no indication for surgery based on LV remodeling) but the presence of PH defined as sPAP >50 mmHg. Guidelines explicitly mentioned that an sPAP >50 mmHg should be confirmed by RHC if this represents the sole criterion for surgery (41).

#### Secondary Mitral Regurgitation

In contrast to primary MR the mitral valve in secondary (or functional) MR is initially structurally normal, and LV dysfunction is initially the result of a process unrelated to the mitral valve (e.g., myocardial infarction with LV remodeling) which then leads to MR. Thus, PAWP elevation and post-capillary PH in patients with secondary MR may be result of LV dysfunction *per se*, MR, and the secondary effects of MR on the ventricle (MR begets MR). In patients with LV dysfunction (LVEF <50%), the severity of secondary MR was identified as an independent predictor of higher sPAP (55). However, a more recent study showed similar mPAP and mPAWP in patients with advanced HFrEF (median LVEF 27%) with no/mild, moderate, or severe secondary MR (56) clearly indicating that MR in this setting is not the only driver of PH. On the other hand, the presence of PH (defined as sPAP ≥45 mmHg by echocardiography) has been shown to be a predictor of death in patients with LVEF ≤40%, but this association between PH and mortality was independent of LVEF, LV diastolic function, and functional MR (57) suggesting a complex pathophysiology of PH in this setting. Although detailed RHC data from a larger population of patients with secondary MR is not available, it is likely that many HFrEF patients with moderate or severe secondary MR and PH have CpcPH rather than simply IpcPH, and that the presence of a pre-capillary component of PH is the mediator of a poor prognosis and the incomplete response or absence of response to a mechanical intervention for MR. This assumption is supported by an important study by Dupont et al. (58) who demonstrated that in patients with HF [*n* = 724, mainly HFrEF (LVEF 19–9%)], low (inframedian) pulmonary capacitance (i.e., a parameter reflecting both high PAWP and high PVR) was associated with poor outcomes. Among the patients with low pulmonary capacitance (*n* = 362, mPAP = 37 ± 9 mmHg, mPAWP = 25 ± 7 mmHg), the prevalence of MR ≥3 + was 43% indicating that there were many patients with secondary MR and CpcPH (58).

A variety of cardiac diseases can be associated with secondary MR. The pathophysiology is therefore variable and can include only moderately dilated LV’s with distorted geometry after an infarct in the territory of right or circumflex artery with tethering of one leaflet and eccentric MR or severely dilated LVs in the context of non-ischemic cardiomyopathy with severe annulus dilatation and tethering of the valve leaflets. As a consequence the mitral valve is not the primary target for therapy in patients with secondary MR and PH. The general approach in these patients is to treat the underlying cardiac disease in an optimal manner.Surgery or interventions for the correction of MR remain an option only for patients remaining symptomatic despite optimal medical and device therapy (41). Percutaneous mitral valve repair in secondary MR has been show to lead to an acute reduction in LAP, mPAWP, and mPAP. However, the baseline mPAP in that study was only mildly elevated (mPAP 29 mmHg), and the reduction of mPAP was relatively modest (- 3 mmHg) (59). In patients with more severe PH (sPAP >50 mmHg) in the context of secondary MR, percutaneous mitral valve repair has been shown to lead to a reduction in sPAP, but sPAP remained higher than in patients without baseline PH, and patients with pre-existing PH had a nearly four-fold higher mortality than patients without (60). In the German Transcatheter Mitral Valve Intervention registry (with two thirds of patients having secondary MR), percutaneous mitral valve repair was associated with a reduction in sPAP from pre-intervention to hospital discharge in patients with sPAP 37–50 mmHg (from 44 to 40 mmHg) and those with sPAP >50 mmHg (from 60 to 51 mmHg) (61). However, there was obviously persisting PH in many patients, and mortality in these two PH groups after percutaneous mitral valve repair was still higher than in those without PH (sPAP ≤36 mmHg) (61). Registry data suggest that there is also some reverse LV and LA remodeling after percutaneous mitral valve repair in patients with secondary MR (62). However, the impact of this phenomenon on PH is unknown.

Given the various determinants of PH in patients with secondary MR and lack of data from controlled studies on the prognostic impact of mitral valve interventions in these patients, current guidelines (41) do not give recommendation regarding valve interventions based on the presence of PH in these patients.

### Aortic Stenosis

**Figure 7:**
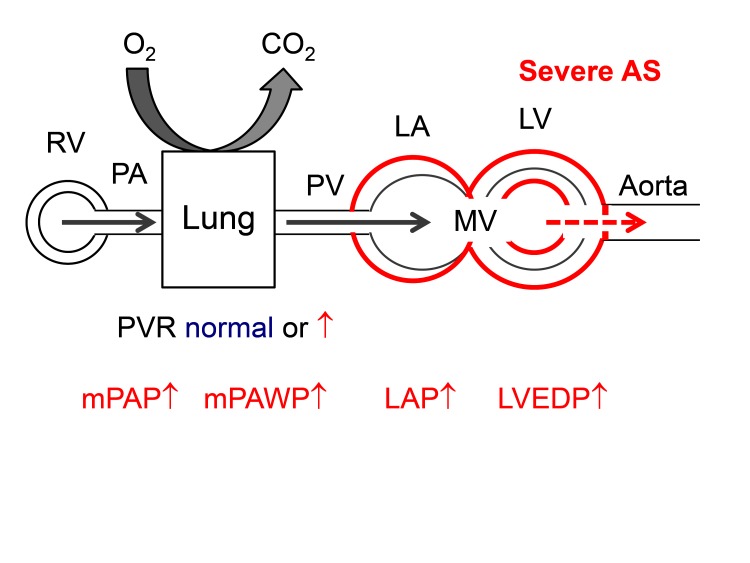
Hemodynamics of severe aortic stenosis (AS). There is pressure overload of the LV with concentric hypertrophy. The LA is secondarily affected by diastolic and systolic LV dysfunction. Abbreviations as in Figure 1.

In severe AS, pressure overload leads to compensatory concentric LV hypertrophy. According to the Laplace equation (LV wall stress ~LV pressure * LV radius/LV wall thickness) an increase in wall thickness in presence of increased LV pressure helps to normalize wall stress. By this mechanism, many patients with severe AS can preserve their stroke volume, i.e., they have a stroke volume index >35 ml/m^2^ and a mean transvalvular gradient >40 mmHg, i.e., high gradient severe AS. However, the process of the development of LV hypertrophy is maladaptive as it is associated with myocardial fibrosis and LV diastolic dysfunction with an increase in LVEDP to achieve a normal LV end-diastolic volume and finally an increase in LAP to ensure LV filling (Figure 7) (63). Thus, in AS with preserved LVEF which represents the vast majority of patients (Table 2), we are faced with a phenotype of HF which is very similar to that seen in patients with HFpEF in whom longstanding hypertension leads to concentric LV remodeling/hypertrophy, diastolic and systolic dysfunction as well as LA dysfunction (64, 65) resulting in a rise in PAWP and PAP already at rest (66) or on exercise (67). Similarly to HFpEF, patients with severe AS can have a spectrum of abnormalities in LV systolic function despite LVEF >50%, and in subset of patients with severe AS and LVEF >50%, stroke volume index is less than 35 ml/m^2^ due to impaired filling and contractility, an entity referred to as paradoxical low flow-low gradient severe AS (68).

As shown in Table 2, PH is common in patients with severe AS. In general there is an association between higher sPAP and more severe AS as expressed by lower aortic valve area (AVA) (14, 28) and lower LVEF (14, 28, 35). However, PH is common also in patients with normal or only mildly reduced LVEF, where LV diastolic dysfunction (17), LA dilatation (23, 28, 35) and dysfunction (69), atrial fibrillation (14), concomitant MR (14, 23) and comorbidities including lung disease (17), obesity (27), anemia (27), and renal failure (27) are key determinants of PH. The majority of studies used echocardiography to assess the presence of PH, and thus the exact hemodynamics c remain unknown. In an interesting RHC study, O’Sullivan et al. (18) found that PH (invasive mPAP ≥25 mmHg) was present in 75% of elderly patients undergoing TAVR, 17% of whom had pre-capillary PH and 83% had post-capillary PH (LVEDP >15 mmHg versus ≤15 mmHg was used for the definition since PAWP was not measured systematically). In the post-capillary PH group, 82% had IpcPH and 18% had CpcPH. Patients with CpCPH had the smallest AVA, the lowest LVEF and the most severe MR.

As shown in Table 2, the vast majority of studies among patients with severe AS undergoing surgical aortic valve replacement (AVR, SAVR) or transcatheter AVR (TAVR) concur that the presence of pre-AVR PH is a predictor of death. A meta-analysis summarizing the data from 16 TAVR studies showed an increased 30 day, 1 year, and 2 year mortality in patients with PH before TAVR (70). The analysis by O’Sullivan et al. (18) revealed that pre-capillary and CpcPH but not IpcPH PH were associated with increased 1 year mortality.

Studies concur that SAVR and TAVR reduced PAP in many but not all patients (18, 20, 28, 29, 31), and that the reduction in PAP is overall relatively modest. In the study by O’Sullivan (18), a reduction in sPAP (measured by echocardiography pre- and post-TAVR) following TAVR was observed in patients with IpcPH (50 to 45 mmHg) and CpcPH (58 to 50 mmHg) PH but not in those with pre-capillary PH (49 to 52 mmHg). The latter is not surprising, and the former is not unexpected either, because PH is the result of the maladaptive changes of the LV and LA in response to pressure overload and only in part due to AS *per se*, and removal of the outflow tract obstruction will not cure the LV disease immediately. A further reduction in PAP over time due regression of LV hypertrophy, LV dysfunction and LA dysfunction following relief of pressure overload may be expected. However, in one study sPAP remained unchanged from one week to one year after TAVR (27), and the same has been shown for patients after SVAR. Similarly, Sinning at al. (26) found a significant but limited reduction in sPAP three months after TVR in patients with baseline sPAP 30–60 mmHg (from 39 to 34 mmHg) and those with baseline sPAP ≥60 mmHg (from 66 to 50 mmHg). The extent of PAP reduction after SAVR/TAVR is clinically important since several studies have shown that persistent PH after TAVR determines prognosis, and that the impact of post-TAVR sPAP is even more important than that of pre-TAVR systolic PAP (14, 28). In the study by Masri et al. (28), 67% of a cohort of 407 patients with severe AS undergoing TAVR had PH at baseline (mPAP ≥25 mmHg on RHC), and 25% of the entire cohort had post-TAVR sPAP >45 mmHg on echocardiography, and these patients had a two-fold higher 2 year mortality than patients without baseline PH and those with baseline PH but post-TAVR sPAP <45 mmHg. Baseline determinants of post-TAVR sPAP >45 mmHg included at least moderate MR, atrial fibrillation/flutter, more advanced LV diastolic dysfunction, and larger LA volume index.

Some studies have shown an early improvement of LV relaxation (71, 72) and LA reservoir and conduit function (72) after TAVR, and at least one study has revealed an improvement in peak atrial longitudinal strain and a reduction in LA volumes three months after TAVR (73). However, data on the effect of TAVR on LV relaxation (mediating the early and active phase of mitral inflow; e’) are controversial (72, 73), and even more importantly the process of recovery of LV stiffness (mediating the late and passive phase of mitral inflow) following AVR is very slow and often incomplete [recently summarized by Kampaktsis et a. (63)]. The presence of myocardial fibrosis is the probably key driver of increased LV stiffness and increased LAP and PH, which may explain the persistence of PH in many patients after AVR. Recent research has shown that the presence of any late gadolinium enhancement (marker of fibrosis) in cardiac MRI predicts mortality after AVR (74). Importantly, myocardial fibrosis in patients with AS is not uniform (75), and the pattern of myocardial fibrosis (extracellular volume expansion versus replacement fibrosis) is clinically relevant (76).

The 2017 ESC guidelines give a IIa indication for AVR in asymptomatic patients with severe AS without another indication for surgery but sPAP >60 mmHg (41). The guidelines state that PH should be confirmed by RHC if PH represents to only indication for surgery (41). Notably, this represents a very advanced stage of the disease with significantly worse prognosis than severe AS without PH (19) and low likelihood of complete recovery of LV diastolic and resolution of PH.

### Aortic Regurgitation

**Figure 8:**
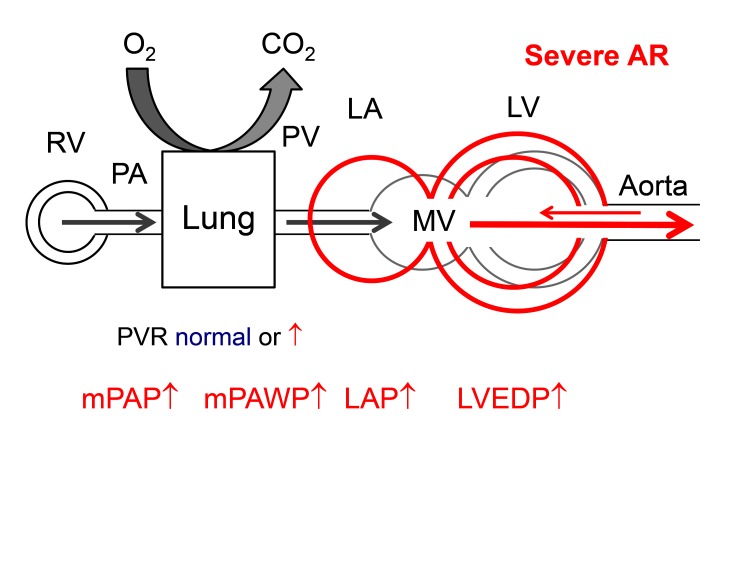
Hemodynamics of severe aortic regurgitation (AS). There is volume overload and dilatation of the LV. The LA is secondarily affected by diastolic and systolic LV dysfunction. Abbreviations as in Figure 1.

Aortic regurgitation imposes combined volume and pressure overload (as indicated by an increased systolic wall stress) (77) to the LV resulting in LV dilatation, eccentric LV hypertrophy, and eventually LV dysfunction. In advanced stages, systolic and diastolic LV dysfunction, LA dysfunction, and secondary MR can lead to increased LAP and post-capillary PH (Figure 8). Markers of this remodeling process (LV end-systolic volume index >25 mm/m2, LVEF <50%) are traditionally used as indications for surgery in asymptomatic patients (41). In contrast to patients with AS, there is little information on the prevalence and prognostic role of PH in patients with AR.

In one large contemporary series, 16% of patients with severe AR had sPAP ≥60 mmHg. These patients had larger LV dimensions, lower LVEF, and more severe MR than those with sPAP <60 mmHg (33). Magne et al. (3) reported mild PH (mPAP 26–35 mmHg) in 23%, moderate PH (mPAP 36–45 mmHg) in 9%, and severe PH (mPAP >45 mmHg) in 5% of patients with various degrees of AR (no details published). In current guidelines (41), the presence of PH is not listed as a criterion for valve replacement in AR given the relative paucity of data on the subject.

### Combined Valve Disease

The combination of several valve lesion is not uncommon the most typical combination being that of AS and MR. The assessment of the severity of AS and MR in this situation is notoriously difficult because MR may lead to a reduction in forward stroke volume and thereby a low flow-low gradient AS situation with all its diagnostic challenges. On the other hand the increased LV afterload due to AS may lead to a reduction of forward stroke volume and an increase in MR respectively. In this context MR is often secondary but primary MR is also possible. Given the detailed discussion above the presence of PH is likely to be common in patients with both relevant AS and relevant MR, and pulmonary pressure may vary depending on filling status, ischemia and dynamic exacerbation of secondary MR, and cardiac rhythm (paroxysmal atrial fibrillation). A detailed discussion of combined valve disease is beyond the scope of the present article. To illustrate the complexity of PH in the context of combined valve disease we just want to mention the study by Toggweiler et al. (78) who have shown that in patients with severe AS undergoing TAVR, concomitant moderate or severe MR at baseline improved in 55% of patients. Predictors of MR improvement included the presence of secondary MR, absence of atrial fibrillation, and absence of PH (defined as systolic PAP > 60 mmHg as assessed by echocardiography or RHC). Thus, PH is clinically highly relevant also in this context although the mechanism underlying this observation is not easy to understand. It may be speculated that RV-LV interactions in presence of significant PH prevent improvements in LV geometry and MR.

### Clinical Implications and Future Directions

The presence of PH is common in valve disease, and most often the mechanism is IpcPH or CpcPH. Thus, PH in valve disease is a marker of HF highlighting that PH is indeed a very important parameter during the assessment and follow-up of patients with valve disease. As discussed above PH is the result of maladaptive changes to the LV, LA, and finally pulmonary vasculature and even the right heart, and these changes may persist fully or in a reduced form after valve intervention resulting in persistent PH with serious prognostic implications. As discussed above the hemodynamic results of valve interventions have mainly been observed by echocardiography. Given the complexity of the hemodynamic pattern and the importance of CpcPH in these patients, systematic RHC early and late after valve interventions may help to better understand their full effects. For instance, we know that aggressive left ventricular unloading by assist device implantation in patients with severe HF and severe CpcPH can reduce not only PAWP but also pulmonary vascular resistance (79).

The replacement or reconstruction of the valve is only one part of the therapy, and a potential residual cardiac dysfunction has to be treated as well. However, the latter aspect of has not been in the focus of research and clinical work in the last years but is only now being recognized. In the patients with a HFrEF phenotype after valve reconstruction or replacement it is relatively evident that standard HFrEF therapies (80) should be established, i.e., angiotensin converting enzyme inhibitors/angiotensin receptor blockers, betablockers, mineralocorticoid receptor blockers, sacubitril/valsartan, ivabradine, and cardiac resynchronization although the effectiveness of these treatments in this context has not explicitly been proven. In patients with a HFpEF phenotype treatment is unknown (80). Given the similarities between HFpEF in the true sense and post-AVR HFpEF spironolactone may be the most attractive drug (81, 82) although this is not proven either. In a substantial number of patients a pre-capillary component of PH may persist after valve replacement although systematic invasive studies in this particular setting are not available. Intense research in the field of PH in the context of HFrEF and HFpEF has revealed that a variety of complex mechanisms are involved in the pathogenesis of CpcPH in these patients, and that specific therapies with proven benefit in group 1 PH may not be efficient in these patients (1). Very recently, the first randomized trial assessing the effect of the 5-phosphodiesterase inhibitor sildenafil on symptoms and cardiac events in patients with persistent PH several months after valve replacement/repair has been published (mPAP ≥30 mmHg required for inclusion; median mean PAP 39 mmHg, median mPAWP 23 mmHg, median pulmonary vascular resistance 3.4 WU) (83). Interestingly, sildenafil therapy was associated with worse clinical outcomes (death, hospital admission, worsening functional class, global symptom burden) than placebo without differences between patient with normal or elevated pulmonary vascular resistance potentially indicating that specific PAH therapies may not be useful for the treatment post-valve replacement IpcPH and CpcPH. This result is very similar to than seen for sildenafil for the treatment of PH (IpcPH and CpcPH) in the context of HFpEF (66).

Given that an exponentially rising number of procedures for valve disease are now being performed also in very sick patients which will improve their symptoms and overall cardiac dysfunction to a certain degree and prevent death due to progressive pump failure, we will be faced with an increasing population of old and sick patients with post-valve replacement/reconstruction heart failure and PH. Thus, intense research in the pathophysiology and the device and medical management of these patients is urgently needed.

## Author contributions

MTM searched the literature, wrote the manuscript, and finalized the manuscript based on the input from the co-authors. All the co-authors critically revised the manuscript for important intellectual content.

## Conflict of Interest Statement

The authors declare that the research was conducted in the absence of any commercial or financial relationships that could be construed as a potential conflict of interest.
